# Investigation of Anti-inflammatory, Antipyretic and Analgesic Activities of *Citrullus colocynthis* in Albino Rats through *in vivo* and Pharmacoinformatics Studies

**DOI:** 10.2174/2772434418666230412105317

**Published:** 2023-11-24

**Authors:** Mubashir Hassan, Nureen Zahra, Amtul Shafi, Saba Shahzadi, Ahmed Moustafa, Andrzej Kloczkowski

**Affiliations:** 1 The Steve and Cindy Rasmussen Institute for Genomic Medicine, Nationwide Children’s Hospital, Columbus, Ohio, 43205, USA;; 2 Institute of Molecular Biology and Biotechnology, The University of Lahore, 54000, Punjab, Pakistan;; 3 School of Psychology, Faculty of Society and Design, Bond University, Gold Coast, Queensland, Australia;; 4 Department of Human Anatomy and Physiology, The Faculty of Health Sciences, University of Johannesburg, Johannesburg, South Africa;; 5 Department of Pediatrics, The Ohio State University College of Medicine, Columbus, Ohio, 43205, USA

**Keywords:** Inflammation, hyperpyrexia, analgesia, α-spinasterol, ascorbic acid, chlorogenic acid, molecular docking, *Citrullus colocynthis*

## Abstract

**Introduction:**

Hyperpyrexia algesia and inflammation are pathological disorders which are treated with synthetic as well as herbal medications.

**Aims:**

The basic aim of the present study is to evaluate the ethnopharmacological activities of phytoconstituents that are present in *C colocynthis* (fruit extract) by using *in vivo* and *in silico* studies.

**Methods:**

Thirty-six albino rats were used in our studies with an average weight between 150-170 g. Anti-inflammatory activity was investigated using carrageenan (an extract from a red seaweed) that induced edema in albino rat paws. However in antipyretic and analgesic activity studies yeast and acetic acid were used to cause pyrexia or algesia respectively. Different doses of acetone fruit extract were used to treat inflammation pyrexia and algesia.

**Results:**

Our results showed that the maximum percentage inhibition of acetonic fruit extract in anti-inflammatory and analgesic activities was observed at 70% and 100% respectively with 400 mg/kg doses and in pyretic activity the maximum inhibitory percentage was 86% with a 100 mg/kg dose. In *in silico* analysis we have shown that bioactive compounds (α-spinasterol ascorbic acid and chlorogenic acid) found in fruit extract have outstanding inhibition properties that involves proteins PTGS2 TLR2 and TRPV4. *C. colocynthis* fruit extract shows results that are statistically significant (*p* < 0.005) and comparable to a reference drug. Acetonic fruit extract of *C. colocynthis* can be used as a natural and safe remedy with no side effects.

**Conclusion:**

Both *in vivo* and *in silico* studies on chlorogenic acid ascorbic acid and α-spinasterol have shown that these are inhibitory compounds that can be used for boosting the immune response.

## 1 INTRODUCTION

Medicinal plants and herbs are used for the treatment of multiple diseases and have been regularly used in medical applications for millennia [1, 2]. *Citrullus colocynthis* (*C. colocynthis*) is a valuable herbaceous plant commonly known as bitter apple or bitter cucumber which belongs to the Cucurbitaceae family [3]. *C. colocynthis* can be found all around the world, such as in East and West Tropical Africa, Northern Africa, Egypt, Asia, and Australia [4, 5].

*C. colocynthis* has angular leaves and fluffy-haired stem and its fruit is smooth, extremely bitter, yellow and has the size of an apple [6]. Bitter apple produces 15 to 30 fruits that are spherical ranging from 7 to 10 cm in diameter [3]. It has been demonstrated that most of the pharmacological efficacy of this plant is linked to the fruit and has been applied to the treatment of different diseases [6]. Extracts of immature fruits show different pharmacological activities, that is, antibacterial activity, anti-inflammatory, antipyretic, antioxidant, anti-diabetic, anticancer, immunostimulant, analgesic, and an-ti-hypolipidemic [7, 8]. Phytochemical analysis of *C. colocynthis* uncovers the presence of chemically bioactive compounds such as 2-O-beta-D-glcopyranosyl-cucurbitacin B and 2, 25-di-o-beta-D-glucopyranosyl cucurbitacin L, Isoorientin 3-o-methyl ether, p-Hydroxy benzoic and Caffeic acid that are present in different parts of plant [9]. Phyto-constituents such as α-spinasterol, ascorbic acid and chlorogenic acid have been selected in this study based on their pharmacological activities *i.e.*, anti-inflammatory, antipyretic and analgesic properties [10].

Non-steroidal anti-inflammatory drugs (NSAIDs) are commonly prescribed for the symptomatic relief of pain and fever in children, and their anti-inflammatory effects are useful for juvenile arthritis and musculoskeletal (MSK) disorders [11-13]. Three different target molecules such as Prostaglandin-endoperoxide synthase 2 (PTGS2), Toll-like receptor 2 (TLR2) and Transient receptor potential cation channel subfamily type 4 (TRPV4) are involved in inflammation and fever infections. In inflammatory pathways, PTGS2 is involved in NF-kappa B, arachidonic acid (AA), Vascular endothelial growth factor (VEGF) and tumor necrosis factor (TNF) signaling pathways. PTGS2 is a rate-limiting enzyme that catalyzes the production of prostaglandin (PG) from AA, which plays an inflammatory role and damages the internal environment of cells. AA generally exists in the form of phospholipid in the cell membrane, a variety of stimuli are capable to activate phospholipase A and release AA from the membrane. Free AA converts into PG under the action of PTGS2 and causes inflammation [14].

During infectious diseases, the temperature of the body must be regulated. TLR2 causes the release of cytokines in a different cell type that delivers fever-inducing signals to AH/POA and induces fever [15]. TLR2 is essential for the development of brain-controlled illness responses such as fever [16]. TRPV is a family of transient receptor potential cation channels that contains six subtypes, ranging from TRPV1 to 6. The change in the expression of the TRPV4 is associated with thermal hyperalgesia. It has been demonstrated that inflammation mediates serval secondary messengers, such as protein kinase A (PKA), phospholipase Cβ (PLCβ) and protein kinase C (PKC) which activate TRPV4. Importantly, TRPV4 releases the following pain transmitters: neuropeptide substance P (SP) and calcitonin gene-related peptide (CGRP) in the spinal dorsal horn. TRPV4 might also lead to electroacupuncture (EA) induced analgesia [17]. TRPV4 is a protein that induces writhing in the albino rat [18]. The perspective of the present study is to evaluate the anti-inflammatory, antipyretic and analgesic activities of phytochemicals present in selected plant extract through *in vivo* and *in silico* model.

## 2 MATERIALS AND METHODS

### 2.1 Experimental Section

#### 2.1.1 Sample Collection

*C. colocynthis* fruit was directly collected from field region of Bahawalpur, Punjab, Pakistan. Furthermore, the collected samples were identified by a botanist from the University of Punjab. Albino rats having an average weight of 150-170 g, were purchased from the University of Lahore (UOL) and used for evaluation of anti-inflammatory, analgesic, and antipyretic activities.

#### 2.1.2 Preparation of Extract

*C. colocynthis* fruit was soaked in acetone (polar solvent) in glass bottles. Mixture was shaken gently for 3-4 minutes and then placed for 16 days at room temperature. On the 16^th^ day, the mixture was filtered with Whatman filter paper and collected in Eppendorf tube.

#### 2.1.3 Anti-inflammatory Activity

Carrageenan-induced edema is a well-defined model of acute inflammation [4, 19]. Thirty-six albino rats were divided into three groups, each group containing 12 rats. A group of rats was euthanized with ketamine (150-200 ul dose of each rat) delivered by injection into the lateral tail vein. The 0.1 ml dose of 1% of carrageenan was injected into sub plantar region of the left hind paw for all albino rats, which caused edema. The control (normal) group was medicated with normal saline, a standard group with diclofenac, and an experimental group with acetone fruit extract of varying concentrations of 50, 100, 200, and 400 mg/kg, respectively.

#### 2.1.4 Antipyretic Activity

Albino rats were divided into different groups according to anti-inflammatory activity model. All groups were treated with 2 ml/kg of Brewer’s yeast mixed with normal saline [20]. After 21 hours of injecting yeast into their necks below the nape, hyperpyrexia was induced. Control group was treated with 2 ml of distilled water, the experimental group was treated with different concentrations (50, 100, 200 and 400 mg/kg) of acetone fruit extract and the standard group with paracetamol.

#### 2.1.5 Analgesic Activity

To evaluate the analgesic activity of *C. colocynthis* fruit extract, acetic acid was injected in rats to induce writhing [4, 19]. All the groups were medicated with acetic acid. One hour after injecting acetic acid into the intraperitoneal cavity, the control group was treated with 0.1 ml/kg of distilled water, standard group with diclofenac at varying concentrations (50, 100, 200, and 400 mg/kg) and experimental group with *C. colocynthis* fruit extract at concentrations 50, 100, 200 and 400 mg/kg. The writhing time was measured with the help of stopwatch.

### 2.2 Computational Methodology

#### 2.2.1 Preparation of Target Proteins

The 3D structures of PTGS2, TLR2 and TRPV4 of albino rats (*Rattus norvegicus*) were accessed from UniProtKB/Swiss-Prot database (https://swissmodel.expasy.org/interactive) because protein structures of PTGS2, TLR2 and TRPV4 were not present in Protein Data Bank (PDB) (www.rcsb.org). These proteins are involved in various pathways, including anti-inflammatory, antipyretic and analgesic activities. PTGS2, TLR2 and TRPV4 structures were retrieved from the SWISS-MODEL (https://swissmodel.expasy.org/interactive) with sequence entries P35355, Q6YGU2 and Q9ERZ8 respectively. UCSF Chimera 1.6 [21] was used to minimize the conformational energies of selected protein models and the structures were saved in PDB format with PyMoL [22]. Furthermore, VADAR 1.8 online server was used to calculate the protein architecture and the statistical percentages of α-helices and β-sheets, turns and coils (http://redpoll.pharmacy.ualberta.ca/vadar) and WinCoot was used to analyse the Ramachandran plots [23].

#### 2.2.2 Selection of Phytocompound and Ligand Preparation

A comprehensive literature survey was performed, showing that α-spinasterol, ascorbic acid and chlorogenic acid are all present in *C. colocynthis* fruit [24-26] and confirmed by online database Dr. Duke [10]. α-spinasterol, ascorbic acid, chlorogenic acid and standard drugs (diclofenac and paracetamol) were retrieved from the PubChem database (https://pubchem.ncbi.nlm.nih.gov/) having accession numbers: 5281331, 54670067, 1794427, 3033 and 1983 respectively and downloaded in SDF format. The retrieved phytocompounds were designed in ChemSketch (ACD/ChemSketch, version 2020.2.0, Advanced Chemistry Development, Inc., Toronto, ON, Canada, www.acdlabs.com, 2021) and accessed in PDB format for further docking studies.

#### 2.2.3 Prediction of Ligand Binding Site of Target Proteins

The active sites of PTGS2, TLR2 TRPV4 provide significant information regarding the functionality of mediated signalling pathways. The active binding sites of PTGS2, TLR2 and TRPV4 were predicted by using Depth Residue (http://cospi.iiserpune.ac.in/depth/), an online tool which explores the probability of amino acids involved in the formation of active binding sites. Furthermore, literature data confirms that these residues are good candidates for the binding sites [27].

#### 2.2.4 Receptor Grid Generation and Molecular Docking

The grid generation for the binding pocket is an important step before performing the docking studies. A cubic grid box with specific values of x-axis, y-axis and z-axis for each of the three proteins was assigned. The grid box for PTGS2 with ligands was adjusted at x = 26.5 Å, y = 41.8 Å and z = 13.1Å. Similarly, for TLR2 the binding pocket dimensions were determined by keeping the grid cantered at x = -1.6 Å, y = 32.5 Å and z = -17.0 Å, while the grid centre for TRPV4 was set at x = -134.5 Å, y = 122.7 Å and z = 130.4 Moreover, the exhaustiveness value was fixed for all docking complexes to obtain the finest binding conformational pose of selected compounds. Docking studies were performed by using PyRx, a virtual screening tool [28]. The different docking complexes were analyzed based on binding energy values (kcal/mol) and interactive behaviour such as hydrogen and hydrophobic interactions. The graphical representation of docking complexes was generated by using Discovery Studio and Chimera 1.6 tools, respectively.

## 3 RESULTS

### 3.1 Anti-inflammatory Activity

#### 3.1.1 Carrageenan Induce Edema in Rat Paw

**Fig. (1):**
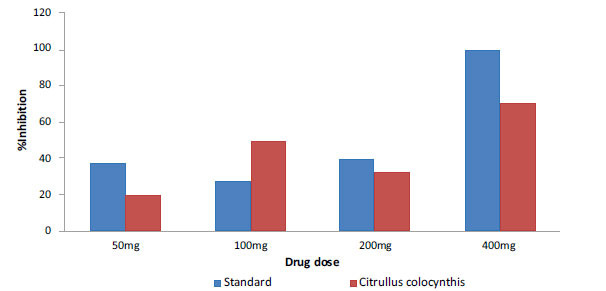
The percentage inhibition of acetone fruit extract of *C. colocynthis* (red) against inflammation in comparison with a standard drug (diclofenac) (blue).

In the anti-inflammatory activity study, the acetone fruit extract of *C. colocynthis* at different concentrations (50, 100, 200 and 400 mg/kg) showed significant results. The maximum inhibition of 70% was observed for a dose of 400 mg/kg. For doses 50, 100 and 200 mg/kg, the achieved inhibition percentages were 20, 50 and 33%, respectively. Similar experiments with doses 50, 100, 200 and 400 mg/kg of a standard anti-inflammatory drug, diclofenac, showed inhibition activities of 37.5, 28.5, 40 and 100%, respectively. All results were statistically significant with *p* < 0.005 (Fig. **1**).

### 3.2 Anti-pyretic Activity

**Fig. (2):**
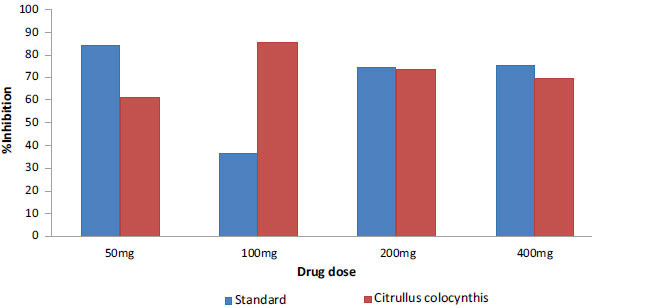
Percentage inhibition of acetone fruit extract of *C. colocynthis* (red) against py-rexia in comparison with a standard drug (paracetamol) (blue).

The acetone fruit extract of *C. colocynthis* shows significant antipyretic activity with *p* < 0.005 at different concentrations of 50, 100, 200 and 400 mg/kg comparable to the standard drug (paracetamol). Percentage inhibition of acetone fruit extract in pyrexia assay, at varying concentrations of 50, 100, 200 and 400 mg/kg were 62, 86, 74 and 85%, respectively. Likewise, the control group showed 0% inhibition after induced pyrexia. The inhibition percentages were 85, 37, 75 and 76% for varying concentrations of paracetamol 50, 100, 200 and 400 mg/kg, respectively (Fig. **2**).

### 3.3 Analgesic Activity

**Fig. (3):**
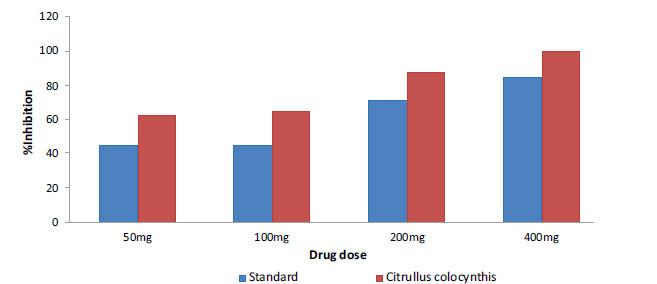
Percentage inhibition of acetone fruit extract of *C. colocynthis* (red) against algesia in comparison with a standard drug (diclofenac) (blue).

The acetone fruit extract of *C. colocynthis* shows statistically significant (*p* < 0.005) analgesic activity. The maximum inhibition of the treated group (100%) was observed for 400 mg/kg dose. For doses with concentration of 50, 100 and 200 mg/kg the observed inhibition percentage was 63, 65 and 88%, respectively. Similar studies performed with a standard analgesic drug, diclofenac, at varying concentrations of 50, 100, 200 and 400 mg/kg, for the standard group showed 45.5 45, 72, and 85% inhibition, respectively. The control group did not show analgesic activity (*i.e.*, 0% inhibition) after injecting acidic acid (Fig. **3**). In Table **1**, the percentages of inhibition of anti-inflammatory, antipyretic and analgesic activities are shown.

### 3.4 Statistical Analysis

Data obtained from rat experiments were statistically analyzed by computing means and standard deviations. Statistical effects were evaluated using ANOVA and all results were considered statistically significant only with *p* ≤ 0.05.

### 3.5 *In silico* Analysis

#### 3.5.1 Protein’s Structure Assessment

PTGS2 belongs to the family of peroxidases and is composed of two chains (A and B) having 604 residues and 69.1 kDa molecular mass, with sequence identity; 87.64%. Protein structure was revealed from UniProtKB/Swiss-Prot database. Furthermore, the structural analysis of PTGS2 by VADAR 1.8 indicated that it consists of 44% of α-helices, 13% of β-sheets, 26% of turns and 42% of the coil. Similarly, the analysis of the Ramachandran plot indicated that 95.99% amino acids were present in the allowed region. TLR2 consists of a single chain (A) containing 704 amino acids and having 89.4 kDa molecular weight. A thorough structural analysis of TLR2 indicated that it consists of 12% of α-helices, 39% of β-sheets, 24% of turns and 47% of coil. Similarly, Ramachandran plot indicates that 90.68% of amino acids are present in the favored region. TRPV4 is comprised of four chains (A, B, C and D) having 871 amino acids with 98.01 kDa molecular weight and it consists of 49% of α-helices, 8% of β-sheets, 24% of turns and 41% of coil. Similarly, Ramachandran plot shows that 94.99% of amino acids are found in the favored region. Fig. (**4**) depicts the Ramachandran graphs of all selected proteins.

### 3.6 Chemistry

α-spinasterol (C_29_H_48_O) is a steroid with a molecular weight 412.7 g/mol, involved in anti-inflammatory and antipyretic activities. Ascorbic acid (HC_6_H_7_O_6_) is water-soluble vitamin with having molecular weight 176.12 g/mol and is involved in anti-inflammatory, antipyretic and analgesic activities [29, 30]. Chlorogenic acid (C_16_H_18_O_9_) is a cinnamate ester obtained by the condensation of trans-caffeic from carboxyl group with quinic acid from 3-hydroxy group and having molecular weight 354.31 g/mol and involved in analgesic activity [31]. Diclofenac (C_15_H_14_O_6_) is a known drug used for anti-inflammatory and analgesic activities. Diclofenac has molecular weight 290.27 g/mol [32]. Paracetamol (C_8_H_9_NO_2_) is a standard drug used in antipyretic activity with molecular weight 155.19 g/mol. The 2D structural representation of these selected phytocompounds has been shown in Fig. (**5**) .

### 3.7 Prediction of Binding Pocket

#### 3.7.1 Active Site Prediction of PTGS2, TLR2 and TRPV4

**Fig. (6):**
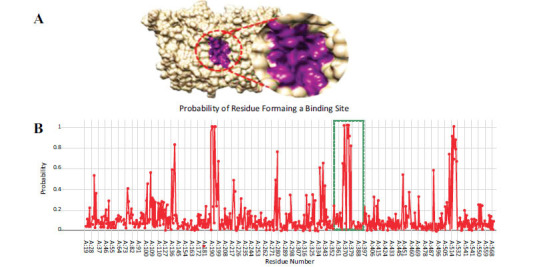
**(A, B).** Active site of PTGS2 attained from Depth Residue tool.

An active site is the functional part of proteins which is involved in catalytic processes and different chemical reactions [33]. (Figs. **6A**,**B**) showed the PTGS2 amino acids to be involved in the formation of the binding site. The Depth Residue results show that the binding site of PTGS2 contains eight different amino amides such as Glu-366, Phe-367, Asn-368, Tyr-371, His-372, Trp-373, His-374 and Leu-377, respectively. The selected amino acids involved in the formation of the binding site have a probability value ranging from 0.81 to 1.0. The results for PTGS2 predict four major peaks in binding regions which indicate the possibility of forming the binding sites. The first peak is observed at Pro-139 having a good probability value 0.82. However, the second peak composed of Gln-189, His-190 and His-192 shows the highest probability value 1.0. The third peak contains 8 amino acids Glu-366, Phe-367, Asn-368, Tyr-371, His-372, Trp-373, His-374, Leu-377 with probability values between 0.64 and 1.0. The fourth peak consists of 7 amino acids Val-509, Ala-513, Phe-515, Ser-516, Leu-517, Lys-518, Gly-519 with probability ranging from 0.72-0.66. The third peak was selected by us as the binding site and these amino acids were also indicated in earlier studies as the active site [28].

The binding site of TLR2 consists of eight residues: Phe-266, Asn-267, Leu-270, Phe-284, Leu-289, Ile-314, Leu-317 and Val-331. These binding site residues have probability values ranging from 0.5 to 1. Figs. (**7A**, **B**) show the surface (**A**) and probability (**B**) of amino acids involved in binding pocket formation. The six active site residue peaks were observed at Phe-266, Phe-284, Leu-289, Ile-314, Leu-317 and Val-331, respectively.

The active site of TRPV4 consists of seven amino acids (Ser-372, Val-385, His-388, His-401, Arg-404, Trp-409 and Asn-425) and the probability values of the selected residues range from 0.2 to 0. 8. Figs. (**8A, B**) shows the surface of TRPV4 (**A**) and amino acids probabilities of forming a pocket (**B**). It consists of six major peaks. The first peak corresponds to Leu-154, the second to Ala-330 with 0.53 probability value, the third to Asp-425 with 0.81 probability value, the fourth to Lys-467 with 0.67 probability value, the fifth and the sixth to Ile 604 and Thr-739 respectively. The amino acids that are present between the second and the third peak have been selected as a binding pocket.

#### 3.7.2 Binding Pockets Analysis and Ligands Interactions

Docking results reveal that all ligands were restricted to the active binding pocket of the target proteins (PTGS2, TLR2 and TRPV4). The superimposition results of all three docking complexes revealed that ligands showed similar conformational behavior and binding interactions as seen from results in Figs. (**9** **-****11**).

#### 3.7.3 Molecular Binding Energy Analysis

PTGS2 docked with the standard drug diclofenac and the bioactive phytocompounds present in the plant (α-spinasterol and ascorbic acid), respectively. The α-spinasterol and Ascorbic acid possessed docking energy of -9.6 and -5.5 kcal/mol, whereas standard diclofenac exhibited -7.3 kcal/mol. The docking results of TLR2 with paracetamol and the plant’s bioactive compounds (α-spinasterol and ascorbic acid) demonstrated that the top docking energy -10.6 kcal/mol was obtained for α-spinasterol, and ascorbic acid showed binding score -5.1 kcal/mol, while paracetamol (standard drug) showed the binding score of -5.9 kcal/mol. The docking results of TRPV4 with paracetamol and the plant’s bioactive compounds (chlorogenic acid and ascorbic acid) showed that the top docking energies of chlorogenic acid and ascorbic acid were -8.6 and -5.8 kcal/mol respectively, while diclofenac (standard drug) showed a binding score of -7.6 kcal/mol (Table **2**).

#### 3.7.4 Binding Analysis of Phytocompounds with PTGS2

**Fig. (12):**
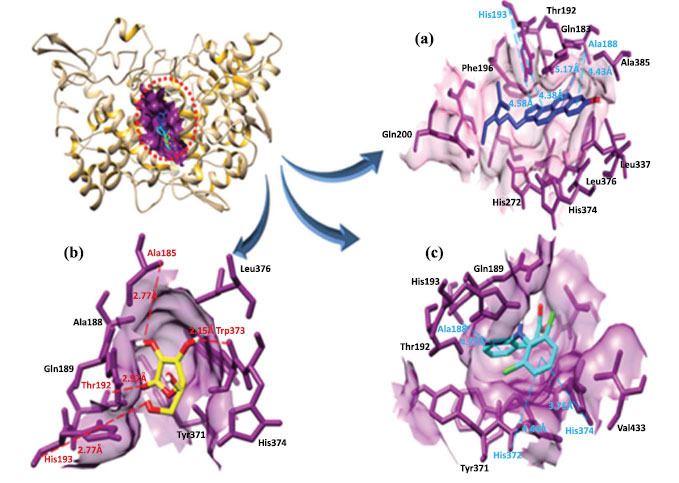
**(a-c).** The binding interaction of PTGS2 with three ligands (blue, yellow and cyan). Target protein (PTGS2) is in tan ribbon line format with a dark magenta surface. Red dotted lines show hydrogen bonds and blue dotted lines indicate hydrophobic interactions and their binding distances in angstrom (Å).

The docking results of α-spinasterol, ascorbic acid and diclofenac against PTGS2 indicated that all drugs bind in the active region of target protein. α-spinasterol formed four hydrophobic bonds at different residues positions, such as His-193, Ala-188 with bond lengths (Å). Likewise, ascorbic acid formed four hydrogen bonds with Ala-185, Try-373, Thr-192 and His-193 and diclofenac formed three hydrophobic bonds with Ala-188, His-372 and His-374 with appropriate binding distances (Table **3** and Fig. **12**).

#### 3.7.5 Binding Analysis of Phytocompounds with TLR2

**Fig. (13):**
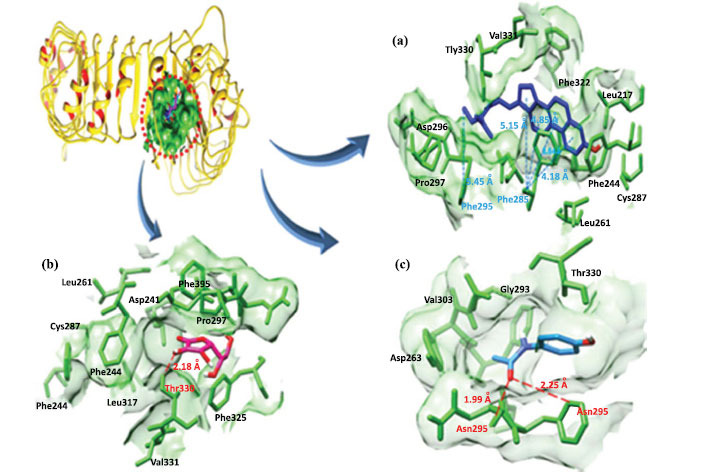
**(a-c).** Binding interaction of TLR2 with three ligands (blue, pink and light blue). Target protein (TLR2) is in yellow ribbon line format with green coloured surface. Red dotted lines show hydrogen bonds and blue dotted lines indicate hydrophobic interactions and their binding distances in angstrom (Å).

Docking results for TLR2 with α-spinasterol showed that six hydrophobic interactions were observed with three residues Phe-295, Phe-266 and Phe-284, respectively. Moreover, ascorbic acid formed one hydrogen bond with Thr-330 and standard paracetamol formed two hydrogen bonds with Phe-295 and Asn-296 (Table **4** and Fig. **13**).

#### 3.7.6 Binding Analysis of Phytocompounds with TRPV4

**Fig. (14):**
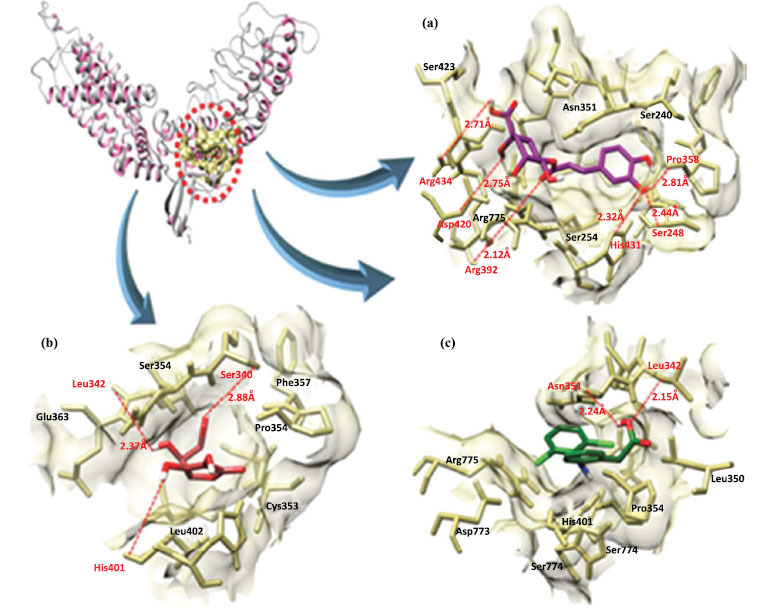
**(a-c).** The binding interaction of TRPV4 with three ligands (purple, red and green). Target protein (TRPV4) is in gray ribbon lines format with tan coloured surface. Red dotted lines show hydrogen bonds and blue dotted lines indicate hydrophobic interactions and their binding distance in angstrom (Å).

The TRPV4-chlorogenic acid docking complex showed that five hydrogen bonds were observed at Pro-358, His-401, Arg-404, Asp-420 and Ser-768, respectively, with appropriate binding distances. Similarly, ascorbic acid formed three hydrogen bonds with His-401, Leu-362 and Ser-360. Likewise, the diclofenac formed two hydrogen bonds with Asp-361and Leu-362 (Table **5** and Fig. **14**). The comparative results showed that all the binding distances were less than 3 Å which enhances the stability of our docking complexes.

The interacted residues comparative analysis showed that most amino acids were common in all docking complexes. The comparative docking analysis showed that four residues Ala188, Gln189, Thr192 and His193 were common in PTGS2 against all drugs. Whereas, in TLR2 and TRPV4 one (Thr330) and two residues (Pro358 and His401) were common in all docking complexes (Table **6**).

## 4 DISCUSSION

Carrageenan induced rat paw edema, pyrexia induced by brewer yeast and writhing caused by acetic acid are used to study anti-inflammatory, antipyretic and analgesic activities, respectively. In the *in vivo* study, acetone fruit extract of *C. colocynthis* showed significant results of anti-inflammatory, antipyretic and analgesic activities in albino rat models. The selected phytocompounds along with standard drugs, also showed good conformational behavior and binding energies (Kcal/mol) in the binding region of the target proteins (*i.e.*, PTGS2, TLR2 and TRPV4), respectively.

The comparison study showed the percentage inhibition of anti-inflammatory, antipyretic, analgesic activities of *C. colocynthis* with respect to dose concentration (Table **1**). They *C. colocynthis* extract with different concentrations of aqueous fruit extract showed comparable results with standard drug. The overall results depicted that with an increase in concentration the percentage inhibition value is also increased in inflammation, antipyretic and analgesic activities, respectively. In the current study, we studied the anti-inflammatory activity of *C. colocynthis* in acetone fruit extract and it demonstrates that acetone fruit extract of 400 mg/Kg shows the highest percentage inhibition that, is 70% and it is statistically significant *p* < 0.05 as compared to standard drug (diclofenac). Whereas, for antipyretic, analgesic activities at same concentration showed 85 and 100%, respectively.

Molecular docking is computational approach which is being employed to check the binding conformational behaviors of ligands against target proteins [34-38]. The active site prediction of target proteins explores the interactive part and core residues which are significantly linked with different functions. The generated prediction results showed that PTGS2 contains eight different amino acids with significant probability values. Similarly, TLR2 and TRPV4 binding pockets explore the core amino acids which might be most potent in the downstream signaling pathways. The predicted docking results justify the good binding potential of ligands (α-spinasterol, Ascorbic acid, Chlorogenic acid, Diclofenac and Paracetamol) against respective molecules PTGS2, TLR2 and TRPV4, respectively (Table **1**). The comparative results showed that α-spinasterol exhibited good energy values as compared to standard drug. The binding interaction profiles of α-spinasterol, ascorbic acid and diclofenac against PTGS2, TLR2 and TRPV4 showed that ligands exhibited within the active target site of proteins and appropriate hydrogen bonding. The overall bonding results showed that the binding distance was comparable with standard value (< 3.5; hydrogen and 5; hydrophobic Å) and enhance the stability behavior of docking complexes (Tables **2**-**5**). The overall results showed that fruit extract of *C. colocynthis* possessed very good therapeutic potential and may be used in future studies to explore novel phytochemicals against inflammatory, pyretic and analgesic activities.

## CONCLUSION

The current research shows that acetonic extract of the fruit of *C. colocynthis* can be used as a natural and safe remedy to cure pain, fever and inflammation with limited side effects. The *in vivo* results showed that fruit extract of *C. colocynthis* exhibited good therapeutical potential against inflammatory, pyretic and analgesic activities. Moreover, *in silico* studies of α-spinasterol, chlorogenic acid and ascorbic acid with PTGS2, TLR2 and TRPV4 receptors indicated good propensity for binding inside all studied target proteins. Our research indicates that the fruit extract of *C. colocynthis* possesses very good therapeutic potential and may be used in future studies to explore novel phytochemicals against inflammatory, pyretic and analgesic activities.

## Figures and Tables

**Fig. (4):**
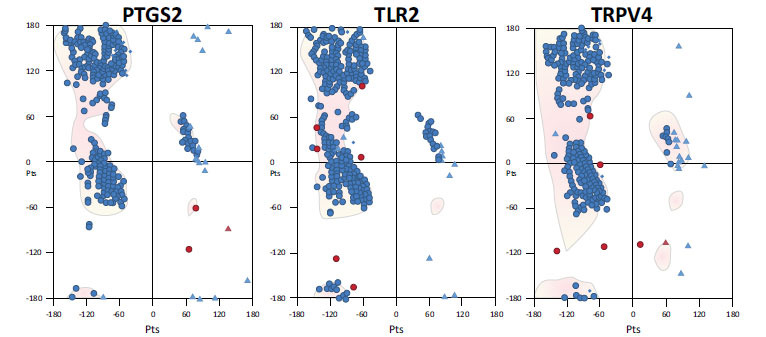
Ramachandran plots of target proteins.

**Fig. (5):**
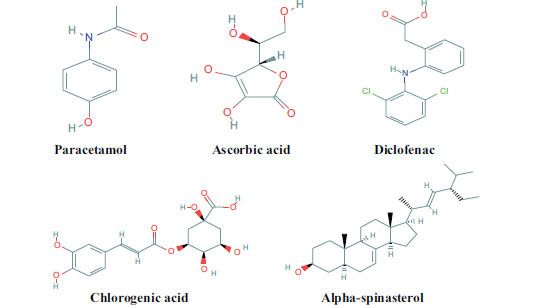
2D structure of selected phyto-compounds.

**Fig. (7):**
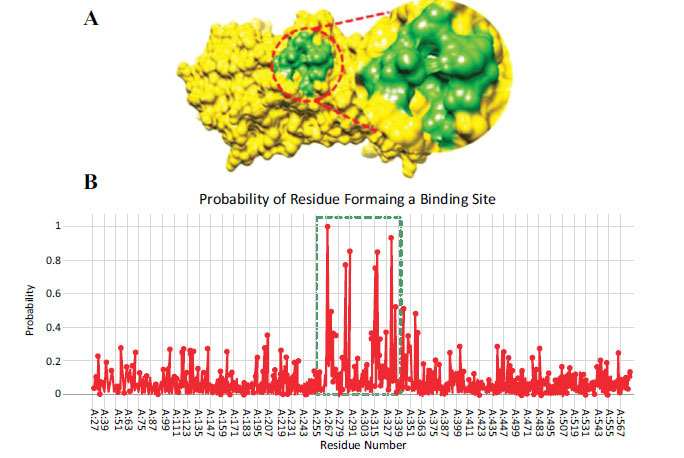
**(A, B).** The binding pocket residues of TLR2 predicted by Depth Residue tool.

**Fig. (8):**
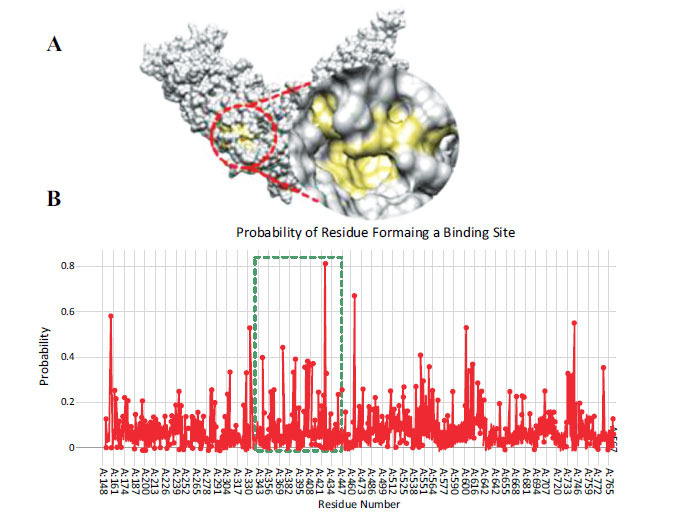
**(A, B).** Binding pocket of TRPV4 predicted by Depth Residue tool.

**Fig. (9):**
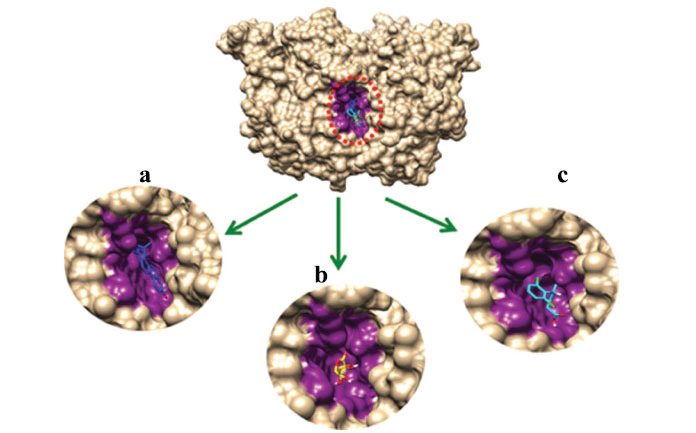
(**a**) α-spinasterol (**b**) Ascorbic acid (**c**) Diclofenac binding conformation with PTGS2.

**Fig. (10):**
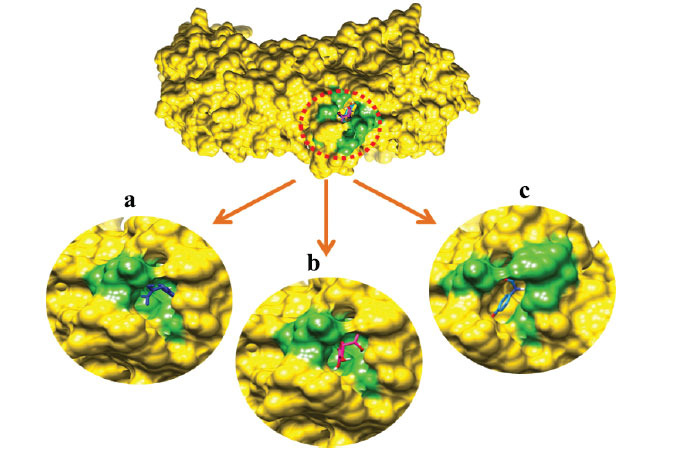
(**a**) α-spinasterol (**b**) Ascorbic acid (**c**) Paracetamol binding conformation with TLR2.

**Fig. (11):**
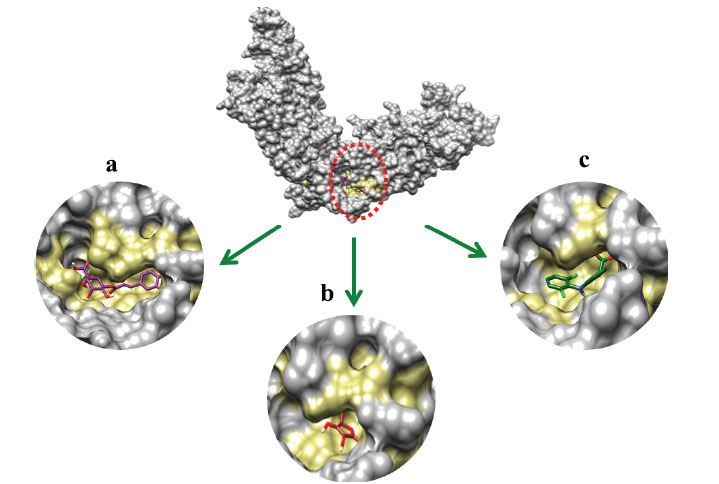
(**a**) Chlorogenic acid (**b**) Ascorbic acid (**c**) Diclofenac binding confirmation with TRPV4.

**Table 1:** Percentage inhibition of anti-inflammatory, antipyretic, analgesic activities of *C. colocynthis.*

**Groups**	**Dose (mg/kg)**	**%Inhibition Anti-inflammation Activity**	**%Inhibition Antipyretic Activity**	**%Inhibition Analgesic Activity**
Control	50	0	0	0
	100	0	0	0
	200	0	0	0
	400	0	0	0
Standard	50	37.5	85	45.5
	100	28.5	37	45
	200	40	75	72
	400	100	76	85
Fruit	50	20	62	63
	100	50	86	65
	200	33	74	88
	400	70	85	100

**Table 2:** Binding energies of target proteins with all the ligands.

**Proteins**	**Binding Energies (Kcal/mol)**				
	**α-spinasterol**	**Ascorbic Acid**	**Chlorogenic Acid**	**Diclofenac**	**Paracetamol**
PTGS2	-9.6	-5.2	-	-7.3	-
TLR2	-10.6	-5.1	-	-	-5.9
TRPV4	-	-5.8	-8.6	-7.6	-

**Table 3:** The nature of bonds between amino acids and ligands within the binding pocket of PTGS2, and bond distances (Å).

**Ligands**	**Amino Acids**	**Bond Distance (Å)**	**Nature of Bond**
α-spinasterol	His-193	4.35 and 4.38	Hydrophobic bond
	Ala-188	5.17 and 5.43	Hydrophobic bond
Ascorbic acid	Ala-185	2.77	Hydrogen bond
	Try-373	2.15	Hydrogen bond
	Thr-192	2.97	Hydrogen bond
	His-193	2.77	Hydrogen bond
Diclofenac	Ala-188	4.77	Hydrophobic bond
	His-372	4.84	Hydrophobic bond
	His-374	3.76	Hydrophobic bond

**Table 4:** The nature of bonds between amino acids and ligands within the binding pocket of TLR2 and their bond distances.

**Ligands**	**Amino Acids**	**Bond Distance (Å)**	**Nature of Bond**
α-spinasterol	Phe-295	5.46	Hydrophobic bond
	Phe-266	5.15 4.65 3.64 4.16	Hydrophobic bond
	Phe-284	5.04	Hydrophobic bond
Ascorbic acid	Thr-330	2.18	Hydrogen bond
Paracetamol	Phe-295	2.25	Hydrogen bond
	Asn-296	1.99	Hydrogen bond

**Table 5:** The nature of bonds between amino acids and ligands within the binding pocket of TRPV4 and their bond distances.

**Ligands**	**Amino Acids**	**Bond Distance (Å)**	**Nature of Bond**
Chlorogenic acid	Pro-358	2.91	Hydrogen bond
	His-401	2.33	Hydrogen bond
	Arg-404	2.70	Hydrogen bond
	Asp-420	2.47	Hydrogen bond
	Ser-768	2.03	Hydrogen bond
Ascorbic acid	Ser-360	2.08	Hydrogen bond
	Leu-362	2.37	Hydrogen bond
	His-401	2.29	Hydrogen bond
Paracetamol	Asn-361	2.24	Hydrogen bond
	Leu-362	2.15	Hydrogen bond

**Table 6:** Common amino acids in docking complexes.

**Ligands**	**PTGS2**	**TLR2**	**TRPV4**
α-Spinasterol	Ala185, Ala188, Gln189, Thr192, His193, Phe196 Gln200, His372 His374, Leu376	Leu261, Phe266 Phe284, Cys287 Pro291, Phe295 Leu317, Phe322 Thr330, Val331	-
Ascorbic acid	Ala185, Ala188 Gln189, Thr192 His193, Tyr371 Trp373, His374 Leu376	Leu261, Asn263 Phe266, Phe284 Pro295, Pro297 Leu317, Phe325 Thr330	Cys353, Ser354 Phe357, Pro358 Ser360, Leu362 Glu363, His401
Chlorogenic acid	-	-	Ser354, Pro358 Ser360, Asn361 Arg392, His401 Arg404, Asp420, Ser423, Ser768 Arg775
Diclofenac	Ala188, Gln189, Thr192, His193, Tyr371, His372, His374, Val433	-	Leu350, Pro358 Asn361, Leu362 His401,,Ser768 Asp773, Arg775
Paracetamol	-	Asp263, Gly293 Asn295, Asn296 Val303, Thr330	-

**Note:** Common residues are highlighted in red color.

## Data Availability

All the data and supportive information is provided within the article.

## LIST OF ABBREVIATIONS

AA: Arachidonic Acid
VEGF: Vascular Endothelial Growth Factor
TNF: Tumor Necrosis Factor
PTGS2: Prostaglandin-endoperoxide Synthase 2
PG: Prostaglandin
TLR2: Toll-like Receptor 2
TRPV: Transient Receptor Potential Cation Channel Subfamily type 4
PKA: Protein Kinase A
PLCβ: Phospholipase Cβ
PKC: Protein Kinase C
EA: Electroacupuncture
CGRP: Calcitonin Gene-RELATED Peptide

## References

[1] Meena M.C., Meena R.K., Patni M.P. (2014). Ethnobotanical studies of Citrullus colocynthis (Linn.) Schrad. An important threatened medicinal herb.. Faslnamah-i Giyahan-i Daruyi.

[2] Fitzgerald M., Heinrich M., Booker A. (2020). Medicinal plant analysis: A historical and regional discussion of emergent complex techniques.. Front. Pharmacol..

[3] Meybodi M.S.K. (2020). A review on pharmacological activities of Citrullus colocynthis (L.) Schrad.. Asian J Res Rep Endocrinol.

[4] Al-Snafi P.D.A.E. (2016). Chemical constituents and pharmacological effects of Cynodon dactylon- A Review.. IOSR J. Pharm..

[5] Pashmforosh M., Vardanjani R.H., Khodayar M.J. (2018). Topical anti-inflammatory and analgesic activities of Citrullus colocynthis extract cream in rats.. Medicina.

[6] Rahimi R., Amin G., Ardekani M.R.S. (2012). A review on Citrullus colocynthis Schrad.: From traditional Iranian medicine to modern phytotherapy.. J. Altern. Complement. Med..

[7] Marzouk B., Marzouk Z., Fenina N., Bouraoui A., Aouni M. (2011). Anti-inflammatory and analgesic activities of Tunisian Citrullus colocynthis Schrad. immature fruit and seed organic extracts.. Eur. Rev. Med. Pharmacol. Sci..

[8] Kapoor M., Kaur N., Sharma C. (2020). Citrullus colocynthis an important plant in indian traditional system of medicine.. Pharmacogn. Rev..

[9] Hussain A.I., Rathore H.A., Sattar M.Z.A., Chatha S.A.S., Sarker S.D., Gilani A.H. (2014). Citrullus colocynthis (L.) Schrad (bitter apple fruit): A review of its phytochemistry, pharmacology, traditional uses and nutritional potential.. J. Ethnopharmacol..

[10] U.S. Department of Agriculture, Agricultural Research Service. 1992-2016. Dr. Duke's Phytochemical and Ethnobotanical Databases.. https://phytochem.nal.usda.gov/.

[11] Poddighe D., Brambilla I., Licari A., Marseglia G.L. (2018). Ibuprofen for pain control in children.. Pediatr. Emerg. Care.

[12] Ribeiro H., Rodrigues I., Napoleão L. (2022). Non-steroidal anti-inflammatory drugs (NSAIDs), pain and aging: Adjusting prescription to patient features.. Biomed. Pharmacother..

[13] Levy D.M., Imundo L.F. (2010). Nonsteroidal anti-inflammatory drugs: A survey of practices and concerns of pediatric medical and surgical specialists and a summary of available safety data.. Pediatr. Rheumatol. Online J..

[14] Li X.H., Liu Y.R., Jiang D.H. (2020). Research on the mechanism of Chinese herbal medicine Radix Paeoniae Rubra in improving chronic pelvic inflammation disease by regulating PTGS2 in the arachidonic acid pathway.. Biomed. Pharmacother..

[15] Zampronio A.R., Soares D.M., Souza G.E.P. (2015). Central mediators involved in the febrile response: Effects of antipyretic drugs.. Temperature.

[16] Welsch J., Hübschle T., Murgott J. (2012). Fever induction by systemic stimulation with macrophage-activating lipopeptide-2 depends upon TLR2 but not CD36.. Innate Immun..

[17] Lin J.G., Hsieh C.L., Lin Y.W. (2015). Analgesic effect of electroacupuncture in a mouse fibromyalgia model: Roles of TRPV1, TRPV4, and pERK.. PLoS One.

[18] White J.P.M., Cibelli M., Urban L., Nilius B., McGeown J.G., Nagy I. (2016). TRPV4: Molecular conductor of a diverse orchestra.. Physiol. Rev..

[19] Rani A., Goyal A., Arora S. (2017). A brief review on Citrullus colocynthis-bitter Apple.. Arch Curr Res Int.

[20] Reddy V.P., Sudheshna G., Afsar S.K. (2012). Evaluation of the antipyretic activity of Citrullus colocynthis fruit extract against yeast induced pyrexia model in wistar rats.. J. Pharm. Res..

[21] Pettersen E.F., Goddard T.D., Huang C.C. (2004). UCSF Chimera? A visualization system for exploratory research and analysis.. J. Comput. Chem..

[22] DeLano W.L. (2002). Pymol: An open-source molecular graphics tool.. Adv. Bio Chem..

[23] Gopalakrishnan K., Sowmiya G., Sheik S.S., Sekar K. (2007). Ramachandran plot on the web (2.0).. Protein Pept. Lett..

[24] Ahmed M., Qin P., Ji M., An R., Guo H., Shafi J. (2020). Spinasterol, 22,23-dihydrospinasterol and fernenol from Citrullus colocynthis L. with aphicidal activity against Cabbage Aphid Brevicoryne Brassicae L.. Molecules.

[25] Nwaogu L.A., Igwe C.U., Ibegbulem C.O., Udebuani A.C. (2017). Effect of seed pulp aqueous extracts of Citrullus colocynthis on oxidative stress parameters of albino rats.. NISEB J.

[26] Hussain A.I., Rathore H.A., Sattar M.Z.A. (2013). Phenolic profile and antioxidant activity of various extracts from Citrullus colocynthis (L.) from the Pakistani flora.. Ind. Crops Prod..

[27] Amaravani M., Prasad N.K., Ramakrishna V. (2012). COX-2 structural analysis and docking studies with gallic acid structural analogues.. Springerplus.

[28] Dallakyan S., Olson A.J. (2015). Small-molecule library screening by docking with PyRx.. Methods Mol. Biol..

[29] Sorice A., Guerriero E., Capone F., Colonna G., Castello G., Costantini S. (2014). Ascorbic acid: Its role in immune system and chronic inflammation diseases.. Mini Rev. Med. Chem..

[30] Adedapo A.A., Falayi O.O., Oyagbemi A.A. (2015). Evaluation of the analgesic, anti-inflammatory, anti-oxidant, phytochemical and toxicological properties of the methanolic leaf extract of commercially processed Moringa oleifera in some laboratory animals.. J. Basic Clin. Physiol. Pharmacol..

[31] dos Santos M.D., Almeida M.C., Lopes N.P., de Souza G.E.P. (2006). Evaluation of the anti-inflammatory, analgesic and antipyretic activities of the natural polyphenol chlorogenic acid.. Biol. Pharm. Bull..

[32] Kantor T.G. (1986). Use of diclofenac in analgesia.. Am. J. Med..

[33] Shanmugam S. (2009). Enzyme technology..

[34] Hassan M., Abbas Q., Ashraf Z., Moustafa A.A., Seo S.Y. (2017). Pharmacoinformatics exploration of polyphenol oxidases leading to novel inhibitors by virtual screening and molecular dynamic simulation study.. Comput. Biol. Chem..

[35] Hassan M, Abbasi MA (2018). Aziz-ur-Rehman , et al. Exploration of synthetic multifunctional amides as new therapeutic agents for Alzheimer’s disease through enzyme inhibition, chemoinformatic properties, molecular docking and dynamic simulation insights.. J. Theor. Biol..

[36] Hassan M., Shahzadi S., Seo S.Y., Alashwal H., Zaki N., Moustafa A.A. (2018). Molecular docking and dynamic simulation of AZD3293 and Solanezumab effects against BACE1 to treat Alzheimer’s disease.. Front. Comput. Neurosci..

[37] Hassan M., Ashraf Z., Abbas Q., Raza H., Seo S.Y. (2018). Exploration of novel human tyrosinase inhibitors by molecular modeling, docking and simulation studies.. Interdiscip. Sci..

[38] Hassan M., Vanjare B.D., Sim K.Y. (2022). Biological and cheminformatics studies of newly designed triazole based derivatives as potent inhibitors against mushroom tyrosinase.. Molecules.

